# First Application of Demand-Triggered Online Adaptive Radiotherapy in the Treatment of Cervical Cancer: A Clinical Report

**DOI:** 10.7759/cureus.69703

**Published:** 2024-09-19

**Authors:** Chenying Ma, Juying Zhou

**Affiliations:** 1 Department of Radiation Oncology, The First Affiliated Hospital of Soochow University, Suzhou, CHN

**Keywords:** artificial intelligence (ai), cervical cancer, fan beam computed tomography, image-guided radiotherapy, online adaptive radiation therapy

## Abstract

Gynecology cancers can reap significant benefits from adaptive radiation therapy (ART) for four major reasons: organ motion, organ deformation, density change, and cavity filling. There are three recognized types of adaptive radiotherapy: offline, online, and real-time. This balance of improved dosimetry versus clinic resources, as well as the optimal timing for adaptations, is still under investigation. The emergence of on-demand online adaptive radiotherapy (OART) can solve the above problems. In this context, we introduce two patients with cervical cancer who used on-demand OART for the first time. One patient with cervical cancer received radical radiotherapy on the United Imaging uCT-ART platform, and another patient with cervical cancer received postoperative adjuvant radiotherapy. The radiotherapy process used OART, which was triggered by senior radiotherapists, assisted by artificial intelligence, and guided by fan-beam computer tomography. Patient 1, who was 54 years old with cervical squamous cell carcinoma, International Federation of Gynecology and Obstetrics (FIGO) stage ⅢC1, underwent radical concurrent chemoradiotherapy. The target volume was reduced in the late stage of radiotherapy. The target volume coverage of the OART plan was better, and the bladder and rectum doses were lower than those of the image-guided radiotherapy plan. Patient 2, who was 56 years old with cervical adenocarcinoma, FIGO stage ⅡA1, underwent postoperative concurrent chemoradiotherapy. If the fractionated treatment during radiotherapy was carried out according to the original plan, treatment off-target would occur, while the OART plan could ensure target coverage. The acute toxic reactions that occurred in both patients during radiotherapy were patient-reported outcome Common Terminology Criteria for Adverse Events 1-2, and no toxic reactions of grade 3 or above occurred. This is the first description of the successful implementation of the uCT-ART-based OART system in EBRT for cervical cancer.

## Introduction

Cervical cancer is a common female malignancy, and radiotherapy plays a key role in the treatment of cervical cancer. However, at present, external beam radiotherapy (EBRT) for cervical cancer faces some difficulties. For example, traditional radiotherapy technology is affected by tumor changes, uterine movement, and bladder/rectum filling and may not be able to achieve accurate dose sculpting, affecting the effect of radiotherapy. Conventional EBRT technology cannot optimize doses and personalize treatment according to individual patient characteristics, which may lead to unsatisfactory efficacy. Tumor radiotherapy resistance or damage to surrounding normal tissues during radiotherapy affects the overall completion of the external beam treatment course [1-3]. Online adaptive radiation therapy (OART) is an emerging technology in radiotherapy. It aims to improve radiotherapy's efficacy and patients' quality of life with individualized, precise, intelligent, and multimodal fusion [4,5]. Recently, a survey of 177 centers from 40 countries showed that 32% of the centers used offline or online adaptive radiation therapy (ART) for cervical cancer EBRT [6]. The application of OART can solve the difficulties faced by EBRT for cervical cancer at this stage and explore a new paradigm for EBRT with reduced toxicity and better efficacy to a greater extent.

Computer tomography (CT)-guided OART for gynecological malignancies is an attractive clinical decision because this imaging modality is widely available in radiotherapy units and requires fewer infrastructure changes. Compared with cone beam computed tomography (CBCT), fan beam computed tomography (FBCT) can make up for the shortcomings of poor soft tissue definition in the latter's imaging [7]. Compared with MRI, FBCT can significantly shorten the duration of the radiotherapy process without increasing the allocation of scarce medical equipment resources [8-10]. In addition, compared with conventional CT, low-dose FBCT uses a lower radiation dose to acquire images, which helps reduce patients' radiation exposure during treatment [11]. In previous research, this research group proposed a complete solution for cervical cancer target area segmentation based on deep learning (DL). The new VB-Net micro-network structure constructed by this solution can compress the model 28 times. The self-developed artificial intelligence (AI) deployment engine saves 75% of hardware resources. It has a multiscale segmentation strategy to improve the segmentation speed, and the actual running speed is <1 second. At the same time, the segmentation accuracy is high, and the clinical target volume (CTV) accuracy reaches 88% [12].

There are three recognized types of adaptive radiotherapy: offline, online, and real-time. The timing of ART initiation can be divided into daily, weekly, or adapt-on-demand. This balance of improved dosimetry versus clinic resources and the optimal timing for adaptations is still under investigation. The emergence of on-demand-triggered OART can solve the above problems. In this case, we used the on-demand-triggered online adaptive radiotherapy technology for the first time and conducted clinical applications in two patients with different types of cervical cancer.

## Case presentation

Patients and tumors

Patient 1, who was 54 years old with cervical squamous cell carcinoma, International Federation of Gynecology and Obstetrics (FIGO) stage ⅢC1, underwent radical concurrent chemoradiotherapy. Patients with locally advanced cervical cancer were treated with weekly sensitization chemotherapy with cisplatin 40 mg/m^2^. Patient 2, who was 56 years old with cervical adenocarcinoma, FIGO stage ⅡA1, underwent postoperative concurrent chemoradiotherapy, and the chemotherapy regimen was platinum-containing doublet chemotherapy.

Informed consent and administrative authorization

Since January 2023, our unit has officially started OART work, including postoperative adjuvant and radical radiotherapy for cervical cancer on uRT-linac 506c (The First Affiliated Hospital of Soochow University, no. 318, 2023). All patients signed informed consent and adhered to the institution's "first use" policy.

OART workflow-triggered on-demand

CT Positioning and Scanning

The patient was scanned during positioning CT and drank 500 mL of water after urination. All patients were positioned by simulated CT using a Philips Brilliance CT Big Bore machine (Eindhoven, The Netherlands) and underwent enhanced CT scanning with a slice thickness of 3 mm. The scanning range was from the upper edge of the 11th thoracic vertebra to 5 cm below the ischial tuberosity. The CT localization image is transmitted to the United Imaging radiotherapy-treatment planning system (TPS), and the TPS built-in AI delineation plug-in automatically segments the region of interest (ROI), namely the treatment target area and the organ at risk (OAR), which is defined as ROI_auto_. The target area modified and reviewed by senior radiotherapists was defined as ROI_edit_. According to the degree of modification, ROI_auto_ was evaluated and classified as excellent (modification degree ≤10%), good (10% < modification degree ≤ 20%), qualified (20% < modification degree ≤ 40%), and unqualified (modification degree > 40%).

Definition of the Target Volume

First, for postoperative adjuvant radiotherapy, according to the target volume delineation guidelines of the Radiation Therapy Oncology Group (RTOG), CTV was defined as the pelvic lymphatic drainage area, including the common iliac lymphatic drainage area, internal iliac lymphatic drainage area, external iliac lymphatic drainage area, presacral lymphatic drainage area, obturator lymphatic drainage area, paravaginal area, and upper vagina (combined with the invasion situation). After surgery, new enlarged lymph nodes in the pelvic area are defined as positive lymph node tumor target volume (gross tumor volume-node, GTVnd). Second, for radical radiotherapy, according to the RTOG and Japan clinical oncology group target volume delineation guidelines, CTV1 was defined as the pelvic lymphatic drainage area, including the primary tumor, cervix, uterus, vagina (combined with invasion), parametrial, common iliac lymphatic drainage area, internal iliac lymphatic drainage area, external iliac lymphatic drainage area, presacral lymphatic drainage area, and obturator lymphatic drainage area. CTV2 was the parametrial area, and positive lymph nodes in the pelvis are defined as GTVnd.

Definition of OAR

The rectum, bladder, sigmoid colon, right colon, small intestine, bilateral femoral heads, pelvis, and spinal cord within the radiation field were defined as OAR tissues.

Definition of Planning Target Volume

All CTV and GTVnd were expanded 5 mm in the six directions of head, foot, abdomen, back, left, and right as planning target volume (PTV).

Prescription Dose

Postoperative adjuvant radiotherapy, PTV1: 45-50.4 Gy/25-28f, planning gross tumor volume-node (PGTVnd) 54-56 Gy/25-28f; radical radiotherapy, PTV1: 45Gy/25f, PTV2: 50 Gy/25f, and PGTVnd: 54-56 Gy/25-28f. The OART plan objectives and dose constraints are detailed in Table 1.

**Table 1:** OART plan objectives and dose constraints ROI: region of interest; OART: online adaptive radiotherapy; PTV: planning target volume; GTVnd: gross tumor volume-node; PGTVnd: planning gross tumor volume-node; OAR: organ at risk

ROIs	Dosimetric requirements	Priority
Targets	Plan objectives	
PTV	D_95 _＞ 100%, D_99 _＞ 95%	2
GTVnd	V_56 _＞ 99%	1
PGTVnd	D_95 _＞ 95%	1
	D_95 _＞ 100%, D_99 _＞ 95%	2
PTV and PGTVnd expansion 5 mm	D_1 _＜ 105%	2
OARs	Dose constraints	Priority
Bladder	D_50 _＜ 50.4 Gy	2
Rectum	D_max _＜ 53 Gy	1
Rectum	D_mean _＜ 40 Gy, D_50 _＜ 50.4 Gy	2
Left femoral head	D_5 _＜ 50.4 Gy	3
Right femoral head	D_5 _＜ 50.4 Gy	3
Small bowel	D_2cc _＜ 54 Gy	1
Small bowel expansion 2 mm	D_max _＜ 53 Gy	1

OART Implementation Workflow

**Figure 1:**
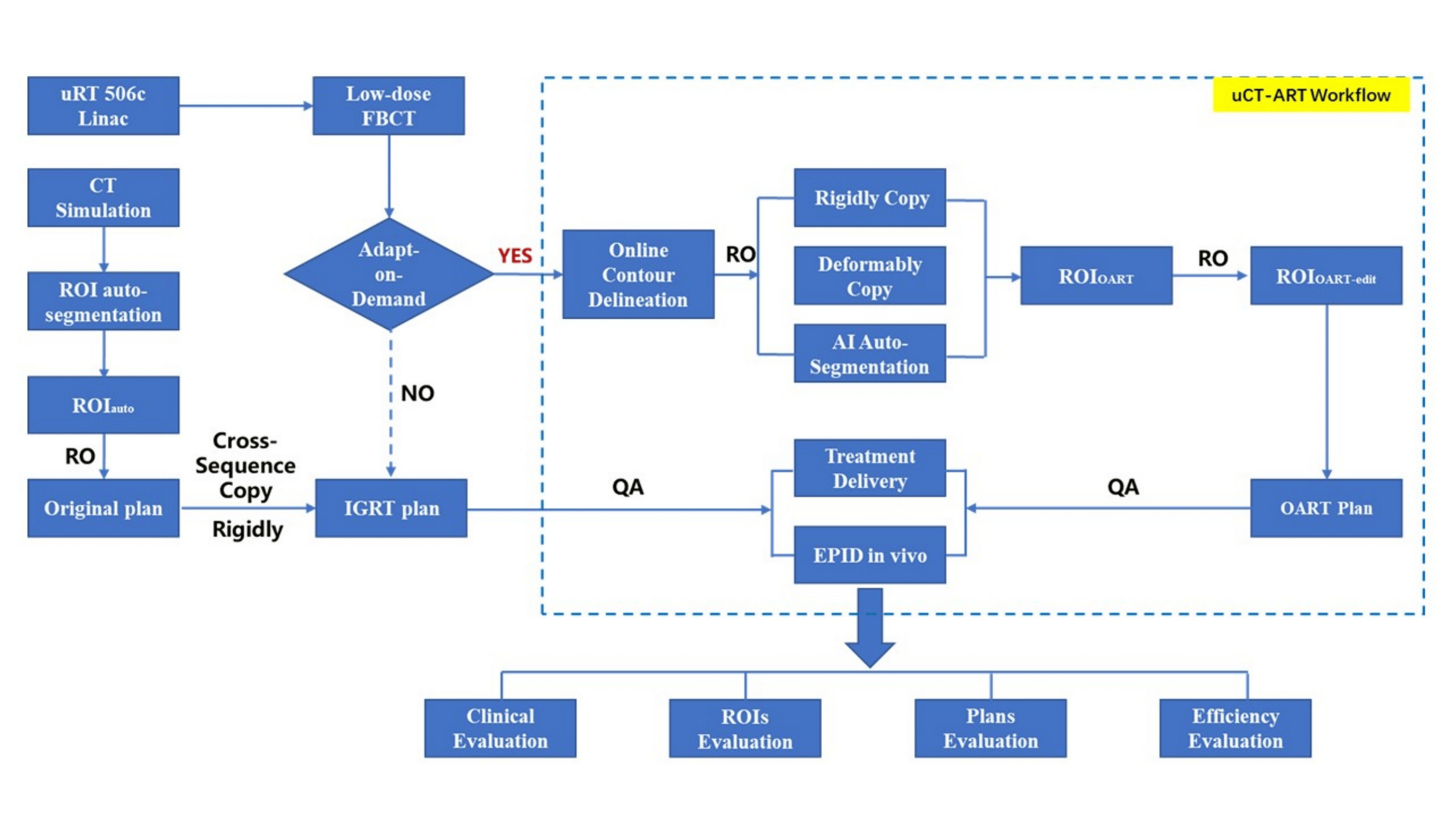
OART execution workflow for cervical cancer CT: computed tomography; RO: radiotherapist; AI: artificial intelligence; QA: quality assurance; ROI: region of interest; OART: online adaptive radiotherapy; IGRT: image-guided radiotherapy; FBCT: fan-beam CT; EPID in vivo: in vivo dose determination based on electronic portal imaging system; ROI_auto_: ROI automatically segmented during positioning CT; ROI_OART_: ROI automatically segmented within OART fractions; ROI_OART-edit_: ROI automatically segmented within OART fractions + ROI modified and reviewed by senior radiotherapists

Volumetric modulated arc therapy was performed using the uRT-linac 506c linear accelerator. Two patients were treated five times a week, once a day. Low-dose FBCT scans were performed before OART every day. Scanning conditions: tube voltage was 120 kV, and tube current was 24 mA. According to the 96th report of the American Association of Physicists in Medicine [13], the abdominal and pelvic tissue weight coefficient was 0.015 mSv/(mGy×cm), and the effective dose of the abdominal and pelvic cavity under low-dose conditions was 0.71 mSv. Image-guided radiotherapy (IGRT) was then performed. The intrafraction FBCT images and positioning CT images were registered. Senior radiotherapists observed the registration results and determined whether OART was triggered. If OART was not triggered, radiotherapy was performed as planned. If it was triggered, the OART process was entered. After entering the OART workflow, you can choose any method of automatic outlining, deformable copying, or rigid copying to generate an ROI outline on the current FBCT image as ROI_OART_. The ROI outline was reviewed, modified, and confirmed by a senior radiotherapist as ROI_OART-edit_. The physicist then created the OART plan instantly. Because the adaptive optimization algorithm refers to the dose distribution of the original plan when calculating the dosimetric parameters, such as controlling the global maximum dose of the adaptive plan to the same level as the original plan, the OART plan generation replicated the original plan to a certain extent. After the plan was generated, the senior radiotherapist and the physicist jointly evaluated the effect of the plan and then approved and scheduled it. The technician performed the beam treatment after the plan was synchronized to the posttreatment delivery application. The specific workflow is shown in Figure 1.

OART Plan Quality Assessment

The original plan was imported into the fractionated FBCT through rigid copying, and the generated plan was called the IGRT plan. The convolution algorithm was used to calculate the dose of the IGRT plan to obtain the dose distribution of the original plan in the fractionated FBCT. The target area average dose, OAR average dose, conformity index, and homogeneity index were used to evaluate the quality of the OART plan and fractionated IGRT plan. Between fractionated treatments, in vivo dose measurement based on the electronic portal imaging system was enabled during beam treatment to monitor the patient's irradiation dose in real time. The plan was considered to have passed when the 2D-γ pass rate was greater than 95% under the 3%/3 mm benchmark.

Clinical Practice

Collect independent timing of each work module in the OART workflow, including ROI modification and review, OART plan optimization, OART plan evaluation, and beam treatment time. In this study, the average total time did not include the setup time, and the average OART adjustment time did not include the setup and beam treatment time. Each fractionated irradiation workflow was participated by one senior radiotherapist, one physicist, and one technician who had received OART workflow training.

Evaluation of adverse reactions to OART radiotherapy

Referring to the Randomized Phase III Study of Standard vs. IMRT Pelvic Radiation for Postoperative Treatment of Endometrial and Cervical Cancer trial (NRG/RTOG 1203) [14,15], the acute gastrointestinal, urinary, and hematopoietic toxic reactions of OART for cervical cancer were evaluated according to the patient-reported outcome version of the Common Terminology Criteria for Adverse Events (PRO-CTCAE) [16]. Data were collected once a week from the first week to the sixth week after the start of radiotherapy.

ROI delineation quality assessment

Two patients had their ROI automatically segmented by the AI-assisted delineation tool on the positioning CT, and the ROI was obtained by ROI_auto_, which was modified and reviewed by senior radiotherapists to obtain ROI_edit_. Given that the baseline-positioning target was processed by ROI_auto_ and the evaluation results were excellent by senior radiotherapists, the target contour segmentation strategy after OART was initiated was to give priority to CTV rigid copy+modification by senior radiotherapists.

Radiotherapy plan quality assessment

The target coverage of the OART plan for the two patients was better than that of the IGRT plan, and the volume of most OARs involved in the IGRT plan was larger than that of the OART plan, reflecting the dosimetric advantage of OART. Table 2 shows the comparison of ROI dose parameters between OART and IGRT in patients with cervical cancer.

**Table 2:** Comparison of dosimetric parameters of ROIs between OART and IGRT in patients with cervical cancer ROI: region of interest; OART: online adaptive radiotherapy; IGRT: image-guided radiotherapy; CTV: clinical target volume; PTV: planning target volume; GTVnd: gross tumor volume-node; PGTVnd: planning gross tumor volume-node; SD: standard deviation ^*^p ＜ 0.05

ROIs	Dosimetric parameters	p value	Δ（OART - IGRT）			
			Average	Min	Max	SD
CTV1	Dmax (%)	<0.001^*^	-0.016	-0.060	0.036	0.015
	Dmin (%)	<0.001^*^	0.042	-0.021	0.473	0.089
	Dmean (%)	0.003^*^	-0.003	-0.024	0.013	0.008
PTV1	Dmax (%)	<0.001^*^	-0.018	-0.060	0.035	0.015
	Dmin (%)	0.001^*^	0.049	-0.159	0.469	0.135
	Dmean (%)	0.029	-0.002	-0.022	0.014	0.008
GTVnd	Dmax (%)	0.184	-0.009	-0.045	0.034	0.022
	Dmin (%)	0.484	<0.001	-0.035	0.050	0.024
	Dmean (%)	0.247	-0.006	-0.037	0.031	0.021
PGTVnd	Dmax (%)	0.202	-0.008	-0.045	0.035	0.023
	Dmin (%)	0.237	0.019	-0.091	0.099	0.060
	Dmean (%)	0.267	-0.006	-0.044	0.036	0.023
Bladder	V100% (cc)	0.388	<0.001	<0.001	<0.001	<0.001
	Dmean (Gy)	0.047^*^	-0.005	-0.067	0.075	0.024
Colon	V100% (cc)	0.363	<0.001	<0.001	<0.001	<0.001
	Dmean (Gy)	0.001^*^	-0.004	-0.037	0.033	0.012
Sigmoid colon	V100% (cc)	0.291	<0.001	<0.001	<0.001	<0.001
	Dmean (Gy)	0.135	-0.002	-0.072	0.036	0.020
Small bowel	V100% (cc)	0.385	<0.001	<0.001	<0.001	<0.001
	Dmean (Gy)	0.037^*^	-0.003	-0.051	0.026	0.014
Rectum	V100% (cc)	0.014^*^	<0.001	<0.001	<0.001	<0.001
	D50% (Gy)	0.049^*^	-0.006	-0.079	0.150	0.035
Left femoral head	D15% (Gy)	<0.001^*^	-0.024	-0.157	0.067	0.047
Right femoral head	D15% (Gy)	<0.001^*^	-0.032	-0.259	0.103	0.064
Spinal cord	Dmean (Gy)	0.029^*^	-0.005	-0.075	0.049	0.024
Pelvis	Dmean (Gy)	0.005^*^	-0.004	-0.040	0.020	0.012

**Figure 2:**
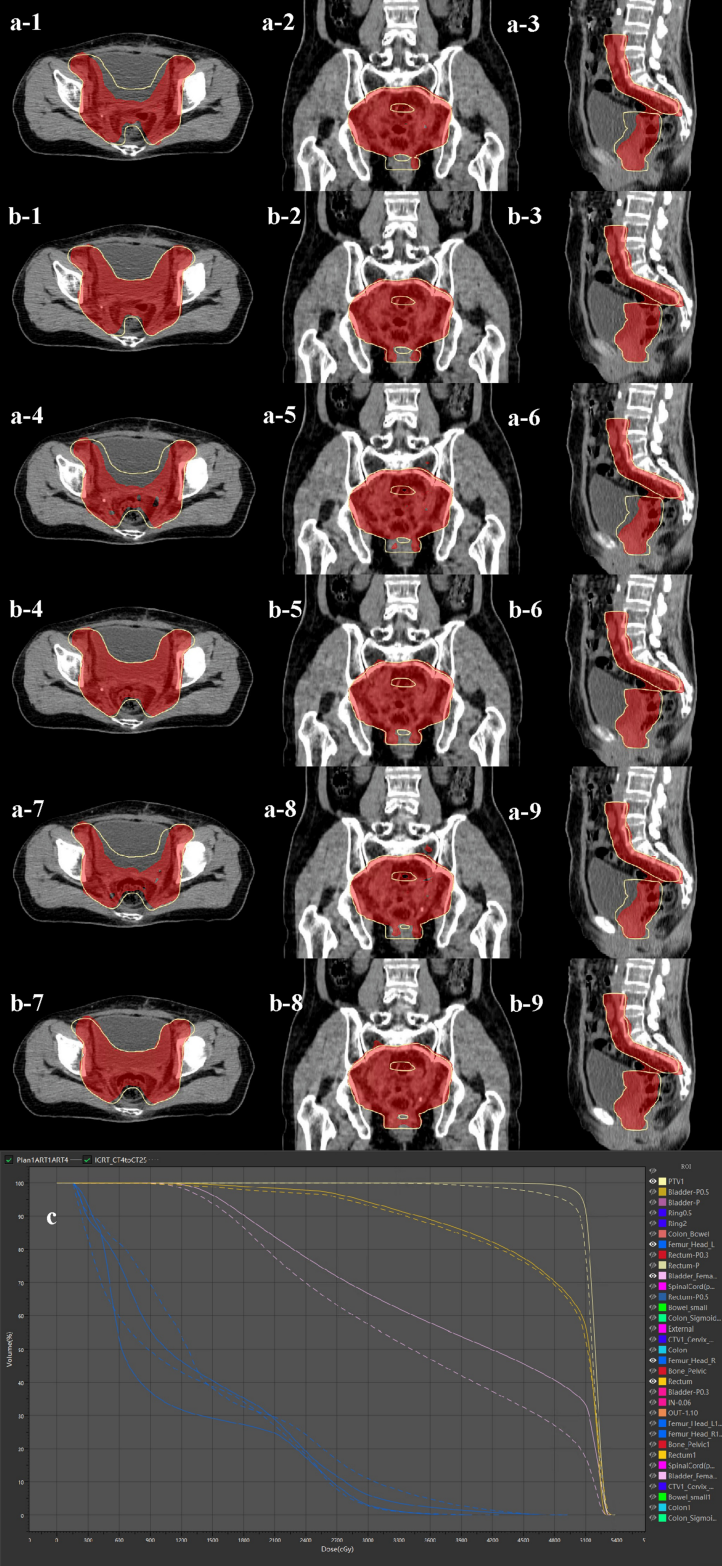
Clinical practice of OART for cervical cancer (case I) Case 1: 56 years old, diagnosed with cervical adenocarcinoma at FIGO II A1 stage. Underwent postoperative synchronous chemoradiotherapy. If the fractionated treatment were carried out according to the original plan, treatment off-target would occur, while the OART plan could ensure target coverage. (a) IGRT plan, (b) OART plan. (c) DVH map. 1,4,7: transverse plane; 2,5,8: coronal plane; 3,6,9: sagittal plane. (a,b) Red blocks were prescription isodose areas, and yellow lines were outlined as PTV. (c) OART plans and dotted lines were IGRT plans OART: online adaptive radiotherapy; IGRT: image-guided radiotherapy; DVH: dose-volume histogram; PTV: planning target volume; CTV1: clinical target volume 1; FIGO: International Federation of Gynecology and Obstetrics

Patient 1 underwent radical synchronous chemoradiotherapy. The target area was reduced in the late stage of radiotherapy. The target area coverage of the OART plan was better, and the bladder and rectal doses were lower than those of the IGRT plan (Figure 2).

Patient 2 underwent postoperative synchronous chemoradiotherapy. If the fractionated treatment during radiotherapy was carried out according to the original plan, treatment off-target would occur, while the OART plan could ensure target coverage (Figure 2).

**Figure 3:**
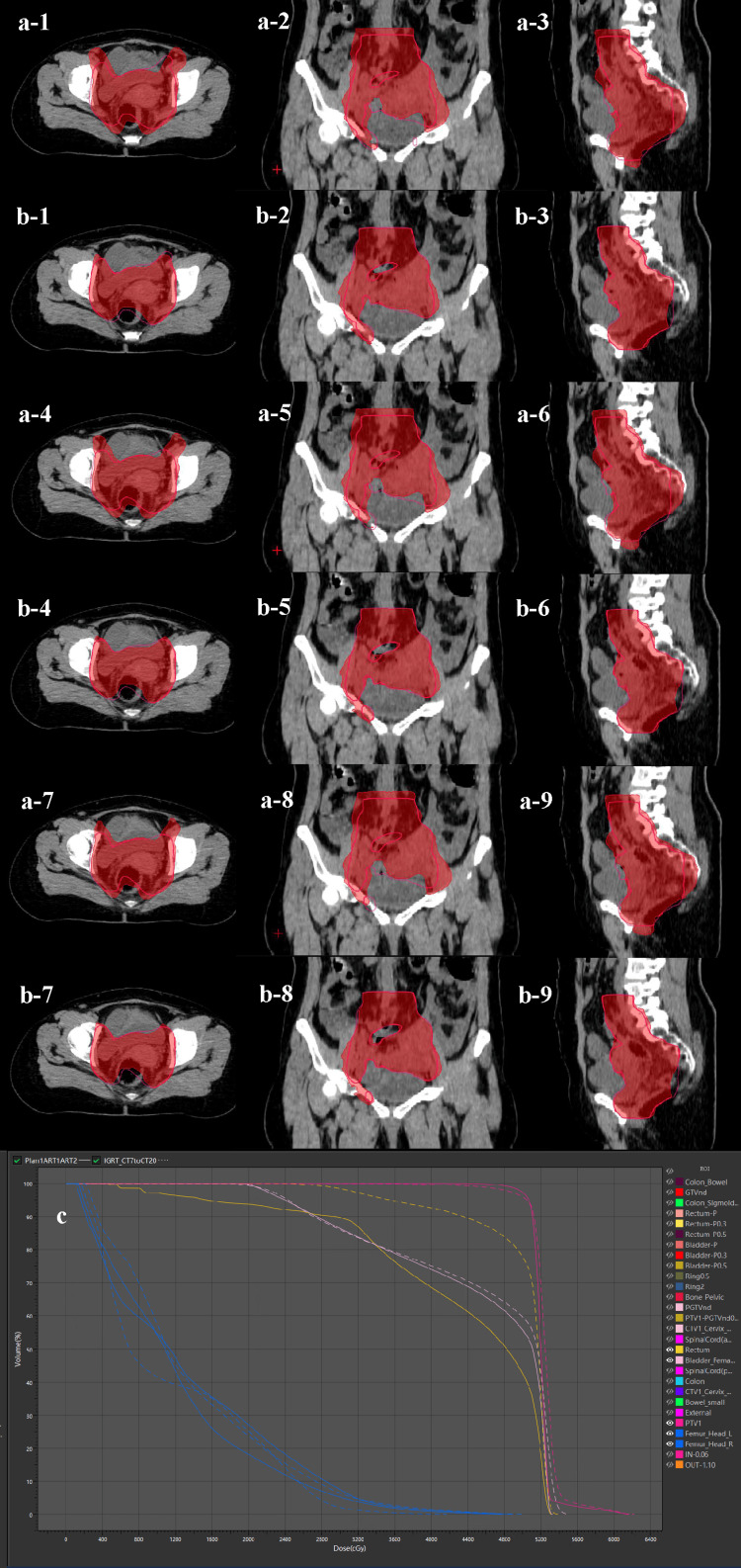
Clinical practice of OART for cervical cancer (case 2) Case 2: 54-year-old and was diagnosed with cervical squamous cell carcinoma at stage FIGO IIIC1. They underwent radical synchronous chemoradiotherapy. During the late treatment period, the target area shrank. The OART plan provided better coverage of the target area than the IGRT plan, and the doses to the bladder and rectum were lower. (a) IGRT plan. (b) OART plan. (c) DVH map. 1,4,7: transverse plane; 2,5,8: coronal plane; 3,6,9: sagittal plane. (a,b) Red blocks were prescription isodose areas, and rose-red lines were outlined as PTV. (c) OART plans and dotted lines were IGRT plans OART: online adaptive radiotherapy; FIGO: International Federation of Gynecology and Obstetrics; IGRT: image-guided radiotherapy; DVH: dose-volume histogram; PTV: planning target volume

Workflow timeliness evaluation

Patient 1 started OART 12 times, with an average total duration of 19.72 minutes for the OART workflow and an average duration of 16.23 minutes for OART adjustment. In different workflow modules, the average durations of ROI modification and review, OART plan optimization, OART plan evaluation, and beam treatment time were 4.25, 3.80, 2.33, and 3.48 minutes, respectively. Patient 2 started OART 15 times, with an average total duration of 15.40 minutes for the OART workflow and an average duration of 12.33 minutes for OART adjustment. In different workflow modules, the average durations of ROI modification and review, OART plan optimization, OART plan evaluation, and beam treatment time were 2.57, 2.57, 1.33, and 3.07 minutes, respectively.

ROI deformation observation

The range of CTV1 volume change was small. Compared with the initial volume in the positioning CT, the relative error of CTV1 volume in different fractions was mainly concentrated in the range of plus or minus 5%, and the maximum value does not exceed 25%. Regarding the changes in the OAR volume between fractions, the volume change range of the bladder, rectum, spinal cord, colon, sigmoid colon, and small intestine fluctuates greatly, and there is no obvious pattern.

OART acute toxicity report

The acute toxicity (including urinary system, digestive system, and hematopoietic system) experienced by both patients during radiotherapy was all PRO-CTCAE grade 1-2, and no grade 3 or higher toxicity occurred.

OART treatment effect

During the one-year follow-up period, both patients had good local control results and quality of life scores. At the same time, no distant metastasis or local recurrence was found.

## Discussion

The application of ART in clinical practice is still in its infancy. According to the timeliness of plan formulation, it can be divided into three types: offline ART [17], online plan library selection [18], and OART [19]. Among them, OART is closer to the actual clinical needs and will become the future development direction of ART. The adaptive radiotherapy scheme selected in this study is a daily plan (a plan of the day, PotD) [20] OART based on FBCT guidance. This treatment strategy fully considers the changes in tumor volume and the daily anatomical changes of the target area and OAR. United Imaging computed tomography-adaptive radiotherapy system provides a full-process technology platform for cervical cancer OART. The platform refines the OART process module, which is divided into four modules: ROI modification and review, OART plan optimization, OART plan evaluation, and beam treatment. In the ROI modification and review module, senior radiotherapists can choose AI to automatically segment the target area and OAR contours on the daily FBCT or choose rigid or elastic registration to copy the ROI. In addition, unlike the daily OART triggering brought about by conventional CBCT-guided PotD [21], this trial process handed over the OART triggering power to senior radiotherapists. On the one hand, it expanded the delineation options of senior radiotherapists more abundantly. On the other hand, it flexibly configured the clinical treatment time and alleviated the shortage of radiotherapy equipment resources.

In the implementation strategy of OART, the first problem to be solved is the contour segmentation problem. The preferred solution is the automatic segmentation of the target area and OAR. Rigaud et al. [22] compared two automatic contour segmentation technologies based on DL methods and verified them on 255 local advanced cervical cancer radiotherapy positioning CT. The results showed that there was good consistency between clinicians and AI contours. In the early stage of this study, a DL tool for automatic contouring of cervical cancer targets based on VB-Net was developed to delineate the CTV of patients undergoing radical radiotherapy and postoperative adjuvant radiotherapy. The results showed that the accuracy of automatic contouring of cervical cancer targets based on DL was comparable to that of manual contouring by senior physicians. Its application in clinical practice will greatly improve work efficiency. Based on the satisfaction with the quality of baseline ROI contouring, CTV rigid copy/OAR automatic segmentation+modification by senior radiotherapy physicians is preferred in the ROI segmentation protocol triggered by OART.

The therapeutic potential of OART is often limited by the large amount of manpower, equipment, time, and other resources. In particular, the OART adjustment time affects the patient's position fixation, the anatomical displacement of organs within the fraction, and other objective factors. Effectively controlling time costs is the key to the successful implementation of OART. Sibolt et al. [23] found that the adjusted OART automatic plan had satisfactory results in the OART treatment of five cases of pelvic malignancies (three bladder cancers, one rectal cancer, and one sarcoma) guided by CBCT, and the median OART adjustment time was 17.6 minutes. In the actual operation of Shelley et al. [24], the average OART time from CBCT acquisition to the start of radiation for five patients with locally advanced cervical cancer, that is, the OART adjustment time, was 29 ± 5 minutes (21-44 minutes). In this study, the average OART adjustment time and the average total time based on FBCT guidance were controlled within 20 minutes, ensuring the high-quality completion of the OART plan while compressing the time cost. At the same time, patients' compliance and tolerance are high, and the team of senior radiotherapists/physicists/technicians are relatively satisfied with the OART process, which lays the foundation for large-scale clinical applications in the future.

## Conclusions

This study describes for the first time the successful implementation of the uCT-ART-based on-demand-triggered OART system in cervical cancer radiotherapy. The OART automatic segmentation contour quality, automatic radiotherapy plan, and treatment duration that meet clinical requirements confirm the feasibility of OART clinical application. On the other hand, the absence of grade 3 or above acute radiotherapy toxicity reactions in the digestive system, hematopoietic system, and urinary system indicates its safety in clinical practice. How to clarify the scope of PTV external radiation reduction, determine the appropriate patient population for OART, optimize the workflow, and meet the requirements of health economics requires a large sample of prospective clinical studies for further in-depth discussion. On the other hand, the on-demand triggered OART process aims to simplify the radiotherapy workflow, making it easier to use and less time-consuming. This includes the development of more efficient planning tools and protocols to reduce the time required for plan adjustments, so that OART can be applied to a wider range of clinical environments, which is one of the future directions of ART development.
